# The effects of short‐term consumption of a Western diet on aerobic exercise performance in bank voles with inherently distinct metabolic rates

**DOI:** 10.1113/EP092646

**Published:** 2025-07-26

**Authors:** Alaa Hseiky, Edyta T. Sadowska, Paweł Koteja

**Affiliations:** ^1^ Institute of Environmental Sciences, Faculty of Biology Jagiellonian University Kraków Poland; ^2^ Doctoral School of Exact and Natural Sciences Jagiellonian University Krakow Poland

**Keywords:** aerobic capacity, endurance exercise, high‐energy diet, obesity, rodent, test to exhaustion

## Abstract

Because obesity and related diseases are partly attributed to unrestricted consumption of a high‐fat, high‐sugar Western diet (WD), several studies have examined its adverse effects on health and physical fitness. However, short‐term WD supplementation has received little attention. We asked whether such supplementation can improve the aerobic exercise performance of animals with inherently distinct aerobic capacity. We used bank voles [*Clethrionomys* (= *Myodes*) *glareolus*] from lines selected for high swim‐induced aerobic metabolism (A) and unselected control lines (C). In a crossover design, we measured endurance running distance, aerobic capacity (${\dot{V}}_{O_{2}\max}$), peak speed (*V*_max_) and respiratory exchange ratio in 98 animals fed standard rodent diet (14.5 kJ/g) or WD (20.5 kJ/g; 48% from carbohydrates, including 28% from added sucrose, and 39% from fat) for 2 days before the trials. The ${\dot{V}}_{O_{2}\max}$, *V*_max_ and endurance distance were higher in A lines than in C lines. In A lines, endurance distance increased with *V*_max_ but not with ${\dot{V}}_{O_{2}\max}$, whereas in C lines it increased more with ${\dot{V}}_{O_{2}\max}$ than with *V*_max_. The WD treatment resulted in increased body mass (*p* = 0.006) and did not affect ${\dot{V}}_{O_{2}\max}$ (*p* = 0.4), but tended to decrease *V*_max_ (*p* = 0.04), endurance distance (*p* = 0.099), mass loss during the trials (*p* = 0.008) and, in A lines only, the respiratory exchange ratio (*p* = 0.02). These results show that the selection experiment provides a model of genetically distinct aerobic athletes and non‐athletes, in whom exercise performance appears to be limited by different mechanisms. Regardless of the genetic background, short‐term WD supplementation does not improve aerobic exercise performance and reduces the effectiveness of exercise as a method of weight loss.

## Abstract (highlights)

- **What is the central question of this study?**
  We asked whether short‐term consumption of a high‐fat, high‐sugar Western diet (WD) can improve the aerobic exercise performance of animals representing a model of genetically distinct ‘aerobic athletes’ and ‘non‐athletes’, i.e., lines of bank voles from an artificial selection experiment.
- **What is the main finding and its importance?**
  Regardless of genetic background, WD provided before exercise did not increase the maximum aerobic exercise metabolism, reduced forced treadmill running endurance and decreased mass loss during exercise. WD consumption before exercise is not a promising exercise enhancer, and it decreases the effectiveness of exercise in combating obesity.

## 1 INTRODUCTION

Obesity and co‐occurring disorders have become a global problem (Phelps et al., 2024). Although many factors could contribute to the epidemics of obesity, the unrestricted consumption of foods high in both fat and sugar, known as the ‘Western diet’ (WD), and reduced physical activity are recognized to be among the main causes (Hruby & Hu, 2015). Therefore, the effects of long‐term WD consumption on the prevalence of obesity and the reduction in cardiorespiratory fitness and aerobic capacity (${\dot{V}}_{O_{2}\max}$), which impair physical performance and exercise capacity, reinforcing the negative metabolic effects of WD, have been studied extensively (Eslami et al., 2020). However, few researchers have asked whether short‐term consumption of a high‐energy diet, such as WD, has similar detrimental effects or whether it could be beneficial in terms of temporarily improving exercise performance. Here, we ask how aerobic exercise performance is affected by short‐term supplementation with a diet rich in both sugar and fat in animals characterized by inherently different aerobic exercise capacity.

The question is also interesting from a sports medicine perspective. Endurance sports, such as long‐distance running, are becoming increasingly popular among amateurs, whereas professional athletes not only try to become faster, but also tend to increase the duration of these challenges (Ronto, 2020). As a result, several approaches have been used to improve endurance performance, including dietary modification (Vitale & Getzin, 2019). This is also the case in non‐human sports disciplines, such as horse racing (Jansson & Harris, 2013). This approach is based on the assumption that performance can be limited by the availability of metabolic substrates, carbohydrates or fats (Burke & Hawley, 2018; Vitale & Getzin, 2019). Consumption of high‐carbohydrate diets can increase glycogen storage, especially in individuals with high aerobic capacity (Areta & Hopkins, 2018), whereas consumption of high‐fat diets can reduce the reliance on endogenous carbohydrate stores and delay their depletion (Burke & Hawley, 2018; Vitale & Getzin, 2019). A high‐carbohydrate diet appears to improve endurance performance more than a high‐fat diet (Erlenbusch et al., 2005). Consumption of a high‐fat, low‐carbohydrate ketodiet increases ${\dot{V}}_{O_{2}\max}$ in high‐intensity interval trained participants, without significantly affecting ${\dot{V}}_{O_{2}\max}$ or time to exhaustion in less intense concurrent or endurance‐trained participants (Cao et al., 2021; Hu et al., 2021; Wang et al., 2022). Similar effects of high‐carbohydrate and high‐fat diets were found in horses (Jansson & Harris, 2013).

Unlike the effects of either high‐carbohydrate or high‐fat diets, the short‐term effects of WD on exercise performance have barely been studied. A systematic search (Table S1) revealed only one such study, which showed that the time to complete a 5 km treadmill run by 11 recreationally active men and women was longer after 4 days of WD consumption than after consumption of a Mediterranean diet (Baker et al., 2019). However, without further research, the generality of the finding remains unknown.

Experimental studies in humans provide direct answers to questions posed from the perspective of human nutrition or sports and clinical medicine, but are subject to several limitations. Factors such as the activity level, training status and the dietary habits of participants before or even during the study period are difficult to control. The genetic background of the participants is usually unknown and cannot be manipulated. Finally, a proper randomization can be difficult to achieve, and the subjects often cannot be completely blinded with respect to the treatment to which they are assigned and to the expected effect of the treatment (Murphy et al., 2021; Wang et al., 2022). Therefore, experiments in animal models, which provide a methodologically sound basis for hypothesis testing, have been crucial to the development of the fundamentals of nutritional and exercise physiology and remain essential for further progress in this research area (Garton et al., 2016).

Results of short‐term dietary manipulations in rodents were similar to those in humans. Rats supplemented with sweet, carbohydrate‐rich cassava tubers experienced an increase in the time to exhaustion during endurance exercise (Yen et al., 2013), whereas rats and mice fed a high‐fat diet had a shorter time to exhaustion and voluntary running distance than those fed a balanced, standard diet (SD) (Murray et al., 2009; Schipke et al., 2019; Xiao et al., 2017). Rats and mice fed long‐term WD either decreased or tended to decrease their time to exhaustion regardless of their training status (Bloomer et al., 2018; Maroofi et al., 2022; Smith et al., 2019). However, similar to human studies, the potential short‐term beneficial effects of WD on exercise performance have not been well studied.

Physical performance is determined by both genetic and environmental factors, and the genetic background can account for a high percentage of the individual variance (Ross et al., 2019). Selection experiments combined with phenotypic manipulation offer a methodologically strong basis to distinguish between the genetic and environmental factors and to analyse the genetic × environment interactions (Ross et al., 2019; Swallow et al., 2009). In several experiments, lines of rodents selected for high or low exercise performance were developed (Koch & Britton, 2001; Swallow et al., 2009), but only in few was the effect of diet on performance studied. Mice from lines selected for high ${\dot{V}}_{O_{2}\max}$ in a swimming trial and from unselected control lines had lower spontaneous physical activity when fed a high‐fat or a high‐carbohydrate diet compared with those fed the SD, and this decrease was not significantly different between the lines (Sadowska et al., 2017). Mice from lines selected for high voluntary wheel running increased activity after a few days of feeding with WD, but the activity was not changed in mice from non‐selected control lines (Acosta et al., 2017; see also Meek et al., 2012, 2010). However, these results relate only to voluntary exercise and cannot be generalized to other exercise types, such as non‐voluntary, forced exercise.

**FIGURE 1:**
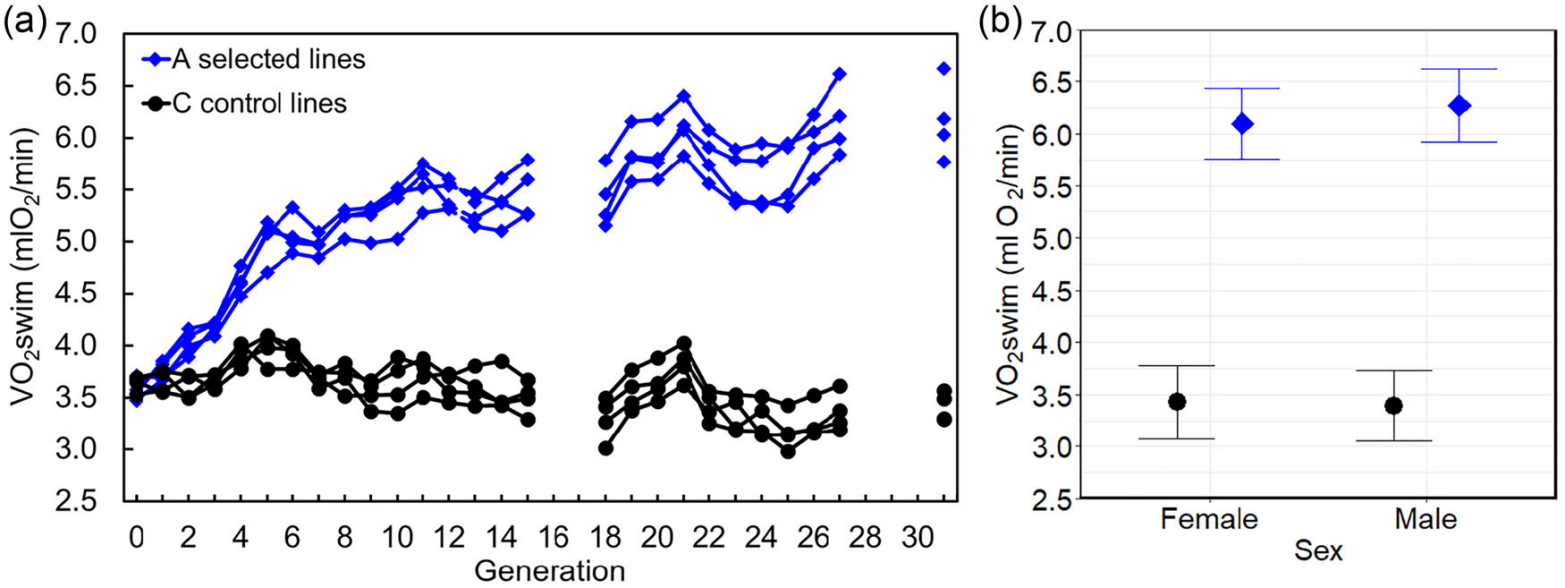
The selection experiment. (a) The direct effect of selection for high 1 min maximum swim‐induced aerobic capacity (${\dot{V}}_{O_{2}\mathrm{swim}}$) in bank voles (replicate line means, not adjusted for body mass; in generations 12, 16–17 and 27–30 the selection was relaxed). (b) Mass‐adjusted ${\dot{V}}_{O_{2}\mathrm{swim}}$ measured in voles from generation 33 at the age of 75–85 days sampled for the present experiment. The ${\dot{V}}_{O_{2}\mathrm{swim}}$ was 6.19 ± 0.3 mL O_2_/min in the A‐line and 3.4 ± 0.3 mL O_2_/min in the C‐line voles (least‐squares means ± 95% confidence interval, adjusted for the mean body mass = 23.81 g, as reported in the analyses of forced running aerobic capacity).

Here, we used bank voles [*Clethrionomys* (= *Myodes*) *glareolus*] from lines artificially selected for high swim‐induced aerobic exercise metabolism (${\dot{V}}_{O_{2}\mathrm{swim}}$; A lines) and unselected control (C) lines (Jaromin et al., 2019; Sadowska et al., 2008). Animals from the A lines achieve >70% higher ${\dot{V}}_{O_{2}\mathrm{swim}}$ than those from C lines (Figure 1), and also have an increase in forced running ${\dot{V}}_{O_{2}\max}$, home cage spontaneous physical activity, basal metabolic rate, daily food consumption and leg muscle mass, but decreased fat content (Jaromin et al., 2019, 2016; Koteja et al., 2009, 2011; Sadowska et al., 2015). Thus, the difference between A‐ and C‐line voles resembles that between human high‐aerobic athletes and non‐athletes (Clemente et al., 2016; Srivastava et al., 2024). Here, we applied a dietary manipulation experiment to learn whether short‐term consumption of WD can enhance ${\dot{V}}_{O_{2}\max}$ and endurance running distance, hence whether these performance traits are limited by substrate availability and whether the effects differ between the aerobic‐athlete and non‐athlete voles.

We expected that WD supplementation prior to the exercise trials could enhance endurance by combining the beneficial mechanisms of high‐fat and high‐sugar diets, such as reduced muscle glycogen utilization, elevated plasma free fatty acid levels, increased β‐oxidative capacity, increased glycogen storage and maintained blood glucose during exercise, which would delay fatigue and prolong performance (Helge et al., 1998; Yen et al., 2013). Importantly, dietary supplementation can increase endurance even if the substrate reserves are not completely depleted (Yen et al., 2013). The ${\dot{V}}_{O_{2}\max}$ is usually assumed to be limited by the factors limiting oxygen delivery or mitochondrial capacity, but factors limiting substrate mobilization and delivery should also be considered (Hoppeler & Weibel, 1998), and some studies have shown that dietary supplementation increased ${\dot{V}}_{O_{2}\max}$ in humans (Hu et al., 2021). Given that the capacity to store substrates increases linearly with body mass, whereas ${\dot{V}}_{O_{2}\max}$ increases allometrically with a slope lower than one (Hoppeler & Weibel, 1998), and given that the length of the oxygen delivery pathway decreases with body size, small animals, such as voles, are more likely to be limited by substrate availability than larger animals or humans. Although ${\dot{V}}_{O_{2}\max}$ is higher in A lines, muscle glycogen stores do not differ between the A and C lines (Jaromin et al., 2016). Thus, A‐line voles can be more limited by the substrate availability than C‐line voles. Therefore, we hypothesized that both A‐ and C‐line voles fed WD for 2 days before the trials would achieve a higher ${\dot{V}}_{O_{2}\max}$ and endurance running distance and that the effect would be more profound in A lines.

## 2 MATERIALS AND METHODS

### 2.1 Ethical approval

The animal colony was under the supervision of a qualified veterinary surgeon. All the procedures performed on animals were approved by the 2nd Local Institutional Animal Care and Use Committee in Krakow, Poland [decision nos 258/2017 (maintenance of the long‐term selection experiment) and 361/2021 (procedures in this experiment)].

### 2.2 Selection experiment

The work was performed on bank voles [*Clethrionomys* (= *Myodes*) *glareolus* Schreber, 1780], a common forest‐dwelling omnivorous rodent widespread in Europe, which has been studied intensively as a model species in ecological research (Boratyński et al., 2020). The wild‐trapped voles were used to establish a selection experiment comprising four replicate lines selected for high swim‐induced aerobic exercise metabolism (A lines) and four replicate lines of control, randomly bred voles (C lines) (Figure 1a; Sadowska et al., 2008). The selection criterion was the maximum 1 min rate of oxygen consumption achieved during an 18 min swimming trial at 38°C (${\dot{V}}_{O_{2}\mathrm{swim}}$), adjusted for differences in body mass and other confounding factors. Details of the experiment rationale and history are presented in our earlier work (Jaromin et al., 2019; Sadowska et al., 2008, 2015).

We measured ${\dot{V}}_{O_{2}\mathrm{swim}}$ with two custom‐built, open‐flow respirometric systems (Koteja, 1996). In most instances, measurements were conducted simultaneously in two animals. Details of the construction of the systems changed across the course of the selection experiment. In generation 19, we used an FC‐10 oxygen analyser and CA2‐2A carbon dioxide analyser in one system and an FC‐10a oxygen analyser (without a carbon dioxide analyser) in the second (Sable Systems, Las Vegas, NV, USA). The 3 dm^3^ respirometric chamber (a cylindrical, transparent glass jar, 15 cm in diameter) was partly filled with water (6 cm space between the water surface and the top of the chamber was maintained), such that the voles could swim freely. The nominal air flow through the respirometric chamber was 2000 mL/min, regulated by a mass‐flow controller (GFC17; Aalborg, Orangeburg, NY, USA; or ERG3000; Beta‐Erg, Warszawa, Poland). A sample of excurrent air was dried first with either a ND2 nafion tube or a DG 1 cold‐trap (Sable Systems), then with a chemical absorber (magnesium perchlorate), and directed to the gas analysers, which recorded the gas concentrations every 1 s. The selection criterion was 1 min ${\dot{V}}_{O_{2}\mathrm{swim}}$ corrected for effective volume (calculated according to appropriate equations in the paper by Koteja, 1996). The water temperature was 38°C to ensure that the animals were using energy only for locomotor activity and not for thermoregulation. We added a drop of dog shampoo to the water to ensure soaking of the entire fur. The measurement was performed in young adults, at the age of 75–95 days. The replicate lines are maintained with 15–20 reproducing families per line. Within‐family selection is applied, and mating between siblings and first cousins is avoided.

### 2.3 Experimental design

The animals were maintained in standard plastic mouse cages (model 1264C or 1290D; Tecniplast, Bugugiatte, Italy) with sawdust bedding, at a constant temperature (20°C ± 1°C), 60% ± 10% air humidity, and 16 h light–8 h dark photoperiod. Water and food (a standard rodent chow, Labofeed H, Kcynia, Poland) were provided ad libitum. The same food was used in the experiment as the standard diet (SD; composition in dry mass according to the producer specification: 25% protein, 43% carbohydrate, 4% fat and 4% fibre; energy, 14.5 kJ/g dry mass, with 31% from protein, 52% from carbohydrates and 12% from fat). The experimental Western diet (WD) diet was prepared by mixing 50% of the powdered standard diet (SD) with 20% butter fat and 30% sucrose (white sugar from sugar beets). The mixture was fixed with starch gel (12.5 g of starch per 1 kg of the mixture) to allow formation of solid pellets. The pellets of WD were then air‐dried at 25°C for 2 days. The WD contained 12% protein, 54% carbohydrate (including 32% from the added sucrose), 20% fat, 2% fibre in dry mass, and 20.5 kJ/g (11% from protein, 48% from carbohydrate, including 28% from the added sucrose, and 39% from fat; computed based on the information on the raw materials). More details about the two diets are presented in Table S2.

For a few reasons, the WD was not supplemented with additional protein, minerals and vitamins, to match the same content as in the SD. First, the SD we used is a breeding type, with high protein (and vitamin and mineral) content; therefore, the protein content in the WD (12%) was not much lower than that in typical maintenance diets used for laboratory rodents [14%–16%; e.g., Maintenance Rat and Mouse Cubes (Specialty Feeds, Glen Forrest, WA, Australia), Teklad Rodent Maintenance Diets (Inotiv, West Lafayette, IN, USA)]. Thus, considering that the single WD treatment lasted only 2 days (Section 2.3, see below), it is unlikely that the animals suffered from a shortage of protein or other essential nutrients. Second, such a diet more closely resembles the typical high‐fat, high‐sugar diets consumed by humans, which are typically not supplemented with additional protein, vitamins or minerals (Cordain et al., 2005). Finally, in a pilot food‐choice experiment, this type of WD was preferred by the voles not only to SD, but also to a protein‐compensated WD produced by the supplier of SD (A. Hseiky, M. M. Lipowska, E. T. Sadowska, and P. Koteja, unpublished data). Thus, especially in the short‐period treatment scheme, it was better to use a diet preferred by the voles to ensure that the animals readily consumed the experimental diet (rather than waiting for a food to which they were accustomed). Both the SD and WD diets were prepared from natural products rather than chemically pure compounds. We recognize that the experiment is therefore not suitable as a basis for making inferences about lower‐level detailed mechanisms underlying the relationship between diet and performance. However, our aim was to focus only on effects at the organismal level, observed in conditions similar to those of normal human life and diet. Importantly, the unknown compounds were the same in both diets, with the exception of those present in the added butter and sugar. Thus, the fact that the diets were prepared from natural compounds does not compromise the methodological correctness of testing the effects of dietary modification. Another experiment, performed on C‐line voles only, showed that the voles fed the WD consumed about the same amount of food, but more energy per day (dry mass: 3.4 g/day; 76 kJ/day) than those fed SD (4.1 g/day; 65 kJ/day; Hseiky et al., 2024).

**FIGURE 2:**
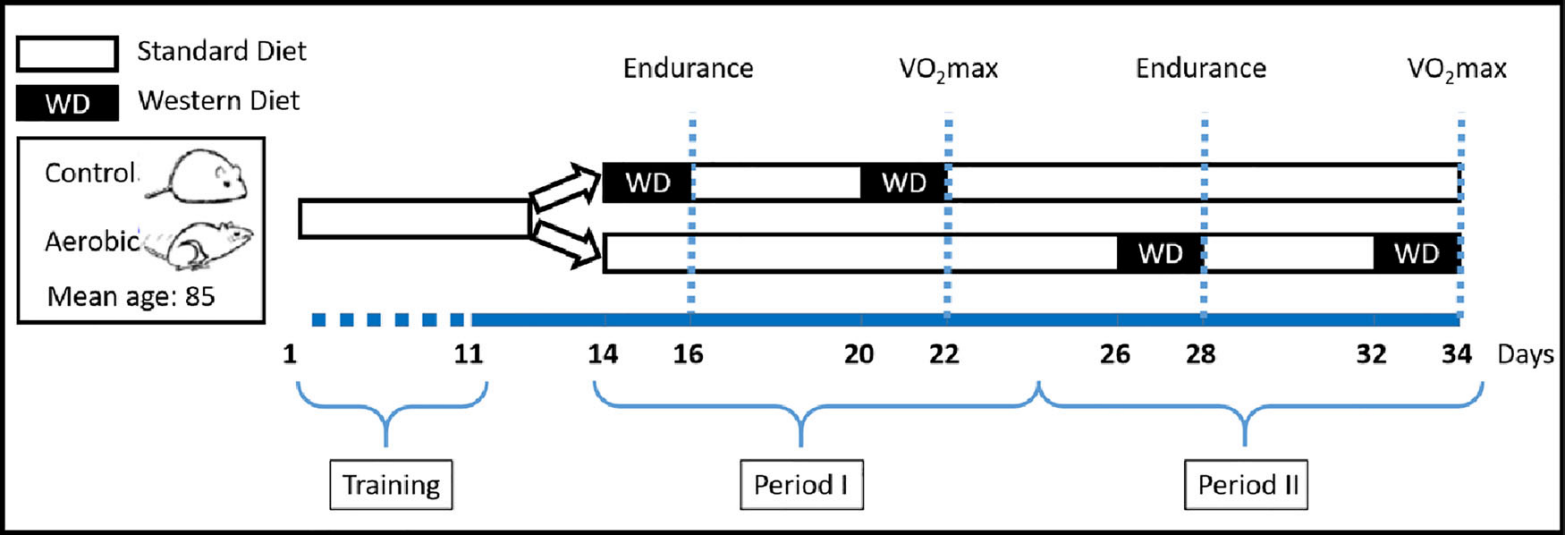
Schematic diagram of the experiment. From day 1 to 10, 96 bank voles were trained three times for each of the two performance traits measurements: endurance running and the maximum forced‐running rate of oxygen consumption (${\dot{V}}_{O_{2}\max}$). Next, the animals were randomly assigned to two crossover design groups. One group received the Western diet (WD) for 2 days before the trials in period I and standard diet (SD) in period II, whereas the other group followed the reverse order. In both study periods, the two performance traits were measured.

The experiment was performed on animals sampled from generation 33 of the selection experiment (Figures 1 and 2). Before the experiment, at the age of 75–85 days, the swim‐induced metabolic rate was measured, in the same way as in the regular selection protocol (Figure 1b). The animals sampled for the experiment achieved the same level of swim‐induced metabolic rates as those in previous few generations (Figure 1b).

At the age of 76–92 days (mean 86 days), 48 males and 48 females were randomly chosen from the A and C lines and placed in individual cages (Figure 2). The number of animals included in the experiment is comparable to that typically used to study the effects of artificial selection on metabolic and locomotor performance traits in rodents (Acosta et al., 2017) and much larger than the numbers used in typical physiological experiments aimed at testing the effects of diet manipulation in rodents (Murray et al., 2009; Xiao et al., 2017). All further procedures were performed on the individually housed animals. At the start of the experiment (the first training; see below), the mean body mass of those animals (mean ± SD; 23.9 ± 4.7 g) was nearly the same as that measured 1–16 days earlier before the preliminary swimming trials (23.7 ± 4.7 g), which indicated a stable metabolic state of the animals. The animals were assigned to six balanced blocks of 16 individuals, on which all procedures were performed on the same day. However, for blocks 1–3 the work was performed on 3 days consecutively, and on blocks 4–6 also on 3 days consecutively, but after a 10 day break. Therefore, in statistical analyses we treated the experiment as consisting of two blocks of 48 individuals.

During the initial 10 days, the animals were trained three times for each of the two performance traits measurements: running endurance and ${\dot{V}}_{O_{2}\max}$. The objective was not to increase the physical performance of the animals, but only to habituate them to the treadmills and entrain them to run regularly. Therefore, the training sessions were shorter and performed at lower speeds than the proper measurements (see Sections 2.4 and 2.5). The training sessions were performed every second day. Before each training session, the animals were weighed, and the average body mass (*M*_b_) was calculated to represent the size of the animal in further statistical analyses. During the training period, the animals were given a small piece of WD three times to avoid an effect of novelty in the main part of the experiment. No animals were excluded during the training period.

On day 11, the animals were randomly assigned to two crossover design groups that differed in the diet they received for 2 days before each of the trial (Figure 2). One group of voles (SW) received SD throughout the entire period I (days 14–22), but in period II (days 26–34) the voles received WD for 2 days preceding each of the two performance trials (days 26–28 and 32–34; on other days they received SD). In the other group (WS), the order was reversed: the animals received WD before the performance trials in period I (on days 14–16 and 20–22) and were fed SD throughout the entire period II. Such a repeated‐measures crossover design ensures both the highest power to test for differences between diets (tested within individuals) and proper control for the possible effect of the order of dietary treatment. In each of the two study periods, two performance characteristics were measured: endurance running on days 16 and 28, and aerobic capacity on days 22 and 34. To avoid a possible experimenter bias, the person performing the measurements was blinded with respect to the experimental group identity of the animals tested. The animals were weighed before and after the measurements, and the mass lost (as a percentage) during the exercise trials was computed. A 6 day break before the subsequent trials allowed the animals to recover fully. The WD diet was given only for 2 days before each trial (Figure 2) to assess whether performance traits were limited by substrate availability even in the absence of metabolic adaptation (similar to in the case of human athletes who consume special meals or take supplements, such as ‘energy drinks’, shortly before a challenging exercise; Erdmann et al., 2021; Ormsbee et al., 2014). However, we could still expect metabolic adaptation to occur after a few days, as suggested by the evidence that fat adaptation can be detected in humans after 5 days of consumption of a high‐fat diet (Burke et al., 2002; Hawley & Leckey, 2015). The remaining WD diet was removed from the cages immediately after each exercise trial and was replaced with SD. We did not measure food consumption during these 2 day periods owing to both methodological and logistical constraints. First, the animals were housed in standard cages with shavings, which made it practically impossible to accurately collect all uneaten food (scraps). In these conditions, short‐term estimates of food consumption are inevitably subject to large relative error and are therefore unreliable. Second, the experiment was highly labour intensive, and the additional workload might have compromised its successful completion. After the measurement of aerobic capacity on day 34, all animals were given SD. Eight days later, they were killed by cervical dislocation and stored at −20°C for a follow‐up study on post‐mortem analysis.

The endurance distance was measured in all animals. The aerobic capacity was measured in all animals except two that were found dead in home cages.

### 2.4 Maximum running endurance

The running endurance was measured using a typical treadmill for humans (ProForm 525ex; iFIT Inc., Logan, UT, USA), modified for this experiment by mounting 30 cm PVC walls to form four parallel tracks 7.5 cm in width, which allowed four individuals to be tested simultaneously, and an interface to DaqFactory Express software (v.16.2; Azeotech Inc., Anchorage, AK, USA), which allowed both automated control of the treadmill speeds schedule and data acquisition (a custom program; P. Koteja). The tracks were 1.1 m long to allow animals temporarily to change running speed without colliding with the back wall of the treadmill. The first 66 cm of the treadmill was covered with an opaque roof to encourage voles to run in the front, darkened part of the tracks (the normal behaviour of voles in open space is an escape to a dark corner). Four ping‐pong balls were placed at the end of each track. The movement of the balls chased and motivated the animals to escape forwards and prevented them from colliding with the back wall of the treadmill. The treadmill was set at 5° slope, which forced a greater running effort at lower speeds.

The protocol of the measurement was similar to that in the studies by Koch and Britton (2001) and Scheer et al. (2018). The speed of the treadmill was set at 1 km/h for the first 2 min, then increased steadily with an acceleration of 0.2 (km/h)/min to reach a speed of 2.6 km/h after 10 min, then with an acceleration of 0.05 (km/h)/min, to reach a maximum speed of 6.1 km/h after 80 min. We applied the protocol with gradually increasing speed to allow comparison of animals with huge differences in endurance capacity. If the speed were set to a constant high value, some animals would not be able to keep up the pace or would become exhausted immediately owing to anaerobic metabolism. On the contrary, if the speed were set to a low value, several animals would run for >1 h, and it would not be possible to complete the measurements according to the assumed schedule. The trial was conducted until the animal exhaustion. When the vole fell between the balls rolling at the end of the treadmill, they were pushed by hand. After three consecutive falls, the vole was considered fatigued and was removed from the track. In our previous study, the maximum speed achieved by C‐line voles in the endurance test conducted with the same protocol (mean ± SD; 3.34 ± 0.46 km/h) was much lower than the maximum sprint speed (7.5 ± 1.41 km/h) (Hseiky et al., 2024). Thus, performance in the endurance test is limited by physiological capacity rather than by biomechanical constraints.

The time, speed and distance at which the vole was removed were recorded automatically. The three values were highly correlated (*r* ≥ 0.99). Therefore, the complete statistical analyses are reported only for the endurance distance, whereas only descriptive statistics for the time to exhaustion and maximum speed are presented in supplementary results (Table S3).

The measurements were performed between 8:47 and 15:40 h at 21.5°C ± 1°C. The proper trial was preceded by three training sessions (Figure 2), lasting 5, 10 and 15 min. In the first training session, the animals were placed on a treadmill at rest for 2 min, followed by 3 min of running at a speed of 1 km/h. In the next two training sessions, the speed was set to 1 km/h for the first 4 min, then increased steadily up to 1.5 km/h.

### 2.5 Maximum forced‐running rate of oxygen consumption (${\dot{V}}_{O_{2}\max}$)

The ${\dot{V}}_{O_{2}\max}$ was measured in a respirometric treadmill for rodents (BTU‐100‐10‐M; Bio‐Sys‐Tech, Białystok, Poland), in a similar manner to our earlier work (Jaromin et al., 2019). The running space was 27 cm × 6.8 cm × 6.5 cm (L × W × H), with a running belt of 21.5 cm, and the rear 5.5 cm electric grid platform. The animals were forced to run by mild electric shocks (0.5 mA, alternating current). The fur of the abdomen and hind legs was moistened with warm water (38°C) to increase electric conductivity (otherwise, the animals ignored the mild electric shocks). To decrease the velocity at which ${\dot{V}}_{O_{2}\max}$ achieved, thereby making the procedure safer, the treadmill was inclined by 6°. We also placed two ping‐pong balls at the end of the moving belt, which helped the animals learn faster to avoid the electric grid. After recording the initial baseline with the treadmill empty for 30 s, the vole was placed in the chamber, and the chamber was closed. The treadmill was started 1 min later at 0.36 km/h and the speed increased by 0.36 km/h each minute (a step exercise test; Scheer et al., 2018). The trial lasted until exhaustion (i.e., until the animal was unable to keep pace with the moving belt; typically, 3–15 min). If the vole attempted to sit on the balls or the electric grid for >3 s, the treadmill direction was reversed momentarily and the balls moved forwards, encouraging the animal to continue running on the belt. If the animals sat on the electric bars twice in a row, the trial was stopped, and the trial duration and maximum speed achieved were recorded (peak treadmill velocity, *V*_max_). After removing the vole from the chamber, the chamber was closed, and after sufficient washout time (∼2 min) a 30 s final baseline was recorded.

The respirometric measurements were performed with an open‐flow, positive‐pressure respirometric system (scheme shown in fig. 10.2A of the paper by Lighton, 2008). Fresh outdoor air was dried with silica gel and pumped through the respirometric chamber of the treadmill at ∼1850 mL/min (STPD, measured before the chamber). The air flow was controlled with mass‐flow controllers (GFC17; Aalborg, Orangeburg, NY, USA), and the exact flow values were determined with high‐precision glass rotameters (ROTA model L2.5/100). A sample of excurrent air (∼200 mL/min) was dried with an ND2 gas sample drier (Sable Systems, Las Vegas, NV, USA) and with chemical absorber (magnesium perchlorate) and directed to FC10 oxygen and CA‐2A carbon dioxide analysers (calibrated according to the producer manuals; Sable Systems, Las Vegas, NV, USA). The oxygen analysers were calibrated at one point against clean, dry air, assuming that it has a concentration of 20.95% oxygen. In the case of the carbon dioxide analyser, the adjustment of zero was performed with respect to carbon dioxide‐free and dry air (carbon dioxide was removed with Ascarite), and the adjustment of span with a reference calibration gas containing 0.8% CO_2_. The gas concentrations were recorded at 1 s intervals through a UI‐2 interface with Expedata software (Sable Systems). The rates of oxygen consumption were calculated with appropriate respirometric equations (supplementary materials in the paper by Sadowska et al., 2015) and corrected for effective volume to achieve instantaneous rates (Lighton, 2008). The ${\dot{V}}_{O_{2}\max}$ was defined as the maximum oxygen consumption in 1 min. The respiratory exchange rate (RER) was calculated at ${\dot{V}}_{O_{2}\max}$ by dividing the rate of CO_2_ produced at the same time by ${\dot{V}}_{O_{2}\max}$.

The ${\dot{V}}_{O_{2}\max}$ measurements were performed between 08:25 and 17:23 h at 21.2°C ± 0.7°C. The proper trial was preceded by three training sessions (Figure 2) lasting 2, 3 and 5 min. The training sessions had the same scheme in speed increase as the main trial, but with lower intensity (the starting speed was 0.24 km/h, and it was increased by 0.24 km/h every minute).

### 2.6 Statistical analyses

The analyses were performed with SAS v.9.4 Mixed procedure (SAS Institute Inc., 2011, Cary, NC, USA), with the Restricted Maximum Likelihood (REML) method of estimation and variance components restricted to positive values. We estimated cross‐nested mixed repeated‐measures ANCOVA models, with three main fixed among‐subject effects: experimental diet, selection direction line type (T) and sex; and the within‐subject fixed effect of the trial number (period). The model included a random effect of replicate line (nested within selection direction). The hierarchical structure of the statistical model (replicate lines nested in selection direction) is required to allow a proper distinction of the effects of selection from random genetic effects, such as genetic drift (Henderson, 1997). To adjust for among‐individual body mass variation and within‐individual changes in the mass across trials, the mean mass in the initial training period (*M*_b_) and the change in body mass (difference between the mass measured before a trial and the initial *M*_b_) were added as fixed covariates. The block (block 1 or block 2), the age and the time of the start of a given measurement were also added as covariates.

Initial models included all possible first‐order interactions between the four fixed categorical factors (diet, T, sex and period), the second‐order interaction diet × T × sex, and the respective random interactions with the replicate line. The models were then reduced by removing non‐significant interactions (*p* > 0.05). However, the T × sex and diet × T interactions were a priori considered biologically meaningful and, together with the respective random line × sex and line × diet interactions, were retained in the models irrespective of their significance. If the interaction between fixed categorical factors was significant, the effect of one of the fixed factors was tested separately for each level of the other (but within the framework of the same model, using the ‘slice by’ option in the LSMEANS command of SAS Mixed procedure). The significance level was corrected for the multiplicity of the tests using Tukey's method.

Similar models were used to analyse body mass. In the analysis of body mass in the training period, diet was replaced by the order group (SW or WS), and the day of the training was included as additional fixed factor. The analysis of body mass measured at the start of the trials included the test type (endurance or ${\dot{V}}_{O_{2}\max}$) as another fixed covariate. The percentage of body mass lost during the trials was analysed separately for the endurance and ${\dot{V}}_{O_{2}\max}$ tests, and with the trial duration as another covariate. The interactions between the trial duration and the main fixed factors (diet, T and sex) were considered to check heterogeneity of the slopes.

The models were fitted initially with either compound symmetry or unstructured residual covariance structure, and we chose the one with the lower Akaike information criterion as the basis for final models. In all the analyses, Satterthwaite's approximation was used to calculate the effective degrees of freedom (d.f.) for *t*‐tests and the denominator d.f. for *F*‐tests (i.e., the d.f. was computed from a combination of the d.f.s of respective random grouping effects and residual term, weighted by the variance contribution of the terms; SAS Institute Inc., 2011). Thus, the d.f.s could take non‐integer values.

To study partial correlations between the endurance distance and ${\dot{V}}_{O_{2}\max}$ or *V*_max_, we used the same linear model as the final, reduced model fitted in the main analysis of endurance distance, but with ${\dot{V}}_{O_{2}\max}$ or *V*_max_ added as another fixed covariate. In initial models, interactions of these covariates with the main fixed factors (diet, T and sex) were also considered to check heterogeneity of the slopes. These models were then reduced stepwise by removing non‐significant interactions. The partial correlation coefficient was assessed separately for A‐ and C‐line voles as a Pearson correlation between the mean residuals (from the two trials of a given individual) obtained in the final models for the three traits of interest.

Likewise, the repeatability of the trials between the two periods was assessed as a Pearson correlation between residuals of the values measured in two trials. The residuals of all the traits were obtained using similar models to those in the main analyses, but separate for periods I and II. To calculate the significance of the partial correlation, we used the same linear model as the final, reduced model fitted in the analysis of period II, but with the period I value as another fixed covariate.

The percentages of body mass lost during both endurance and ${\dot{V}}_{O_{2}\max}$ trials were square root transformed, but the results were presented after back transforming the least‐squares means. Complete tables with descriptive statistics and results of the mixed ANCOVA models are presented in the Supplementary Results (Tables S3–S5). Here, we provide the main results as adjusted least‐squares means with the 95% confidence interval (95% CI).

## 3 RESULTS

### 3.1 Body mass during the locomotor tests

**FIGURE 3:**
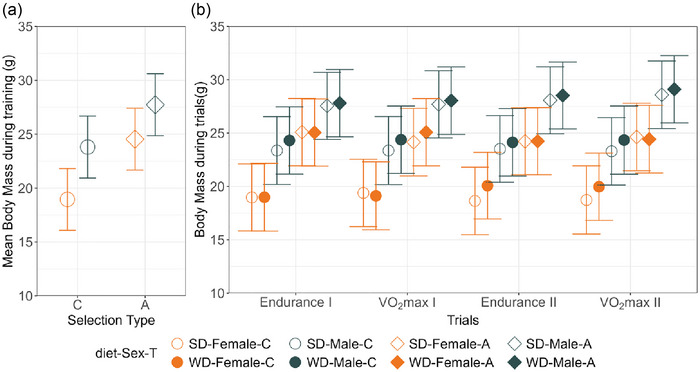
Body mass during the experiment. (a) Average body mass measured during the training period. (b) Body mass measured at the start of the endurance running and maximum forced‐running rate of oxygen consumption in periods I and II (least‐squares means ± 95% confidence limits). On average, males were larger than females, A‐line voles larger than C‐line voles, and the body mass was 0.5 g higher after the animals ate WD than SD. Note that an overlap of the confidence limits does not necessarily indicate that an effect is not significant, because the confidence limits in this figure are based on among‐individual variation for a particular subgroup in particular conditions (diet and type of test), whereas the inferences concerning the effects of sex and selection are based on comparisons averaged across the conditions and groups of other factors, and inferences concerning the effect of diet are based on within‐individual comparisons. See Table S4 for results of the proper significance tests. Abbreviations: A, aerobic lines; C, unselected control lines; SD, standard diet; WD, Western diet.

Males were heavier than females (*p *< 0.0001), and voles from the A lines were heavier than voles from the C lines (*p* = 0.03), and the differences did not change significantly during the experiment (interactions with time variables, *p* ≥ 0.08; Figure 3). The mean body mass at the start of the experiment and throughout the training period (days 1–11; Figure 2) did not differ between voles assigned to the two treatment‐order groups, which indicates a proper randomization (Figure 3a,b). Body mass remained on average nearly stable during the training period (mean body mass change ± SD: −0.3 ± 1.3 g). In the WS group, body mass increased during the first trial period (i.e., when the animals received WD diet before the locomotor trials), but it returned to the initial value during the second trial period (when the animals received SD diet). In the SW group, in which the order of diet treatment was reversed, the temporal pattern of body mass changes was also reversed; body mass did not change in first trial period, but increased during the second trial period. Body mass increased during the first 2 day episode of eating the WD diet (before endurance running tests), but it did not increase further during the next 2 day episode of eating WD (before the ${\dot{V}}_{O_{2}\max}$ tests). Thus, body mass was consistently higher after the animals ate WD than SD (least‐squares means ± 95% CI; SD, 23.7 ± 2.1 g, WD, 24.2 ± 2.1 g; diet, *p* = 0.006).

### 3.2 Locomotor and metabolic traits

**FIGURE 4:**
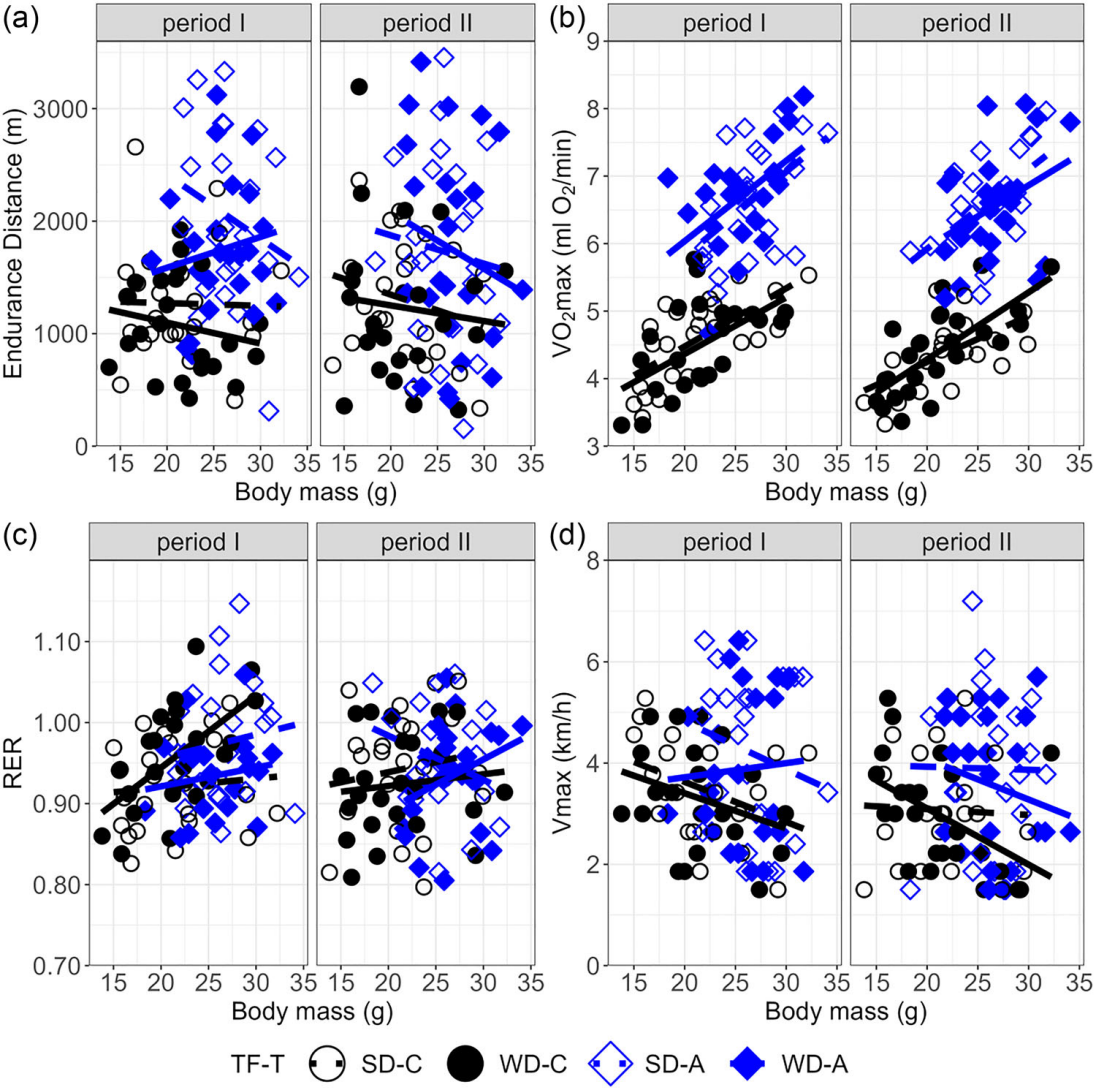
Correlation between locomotor performance and body mass across the two repeated trials. (a) Endurance distance. (b) Forced running aerobic capacity (${\dot{V}}_{O_{2}\max}$). (c) Respiratory exchange ratio calculated at ${\dot{V}}_{O_{2}\max}$. (d) Maximum speed achieved during the ${\dot{V}}_{O_{2}\max}$ trial. Graphs show the raw data. Lines are from linear regression: dotted lines represent SD, while solid lines represent WD. Abbreviations: A, aerobic lines; C, unselected control lines; SD, standard diet; WD, Western diet.

The ${\dot{V}}_{O_{2}\max}$ and the RER increased with body mass (*p *< 0.0001 and *p* = 0.01, respectively) and the change in body mass (*p* ≤ 0.0007), but the maximum speed achieved during the ${\dot{V}}_{O_{2}\max}$ trial (*V*_max_) and endurance distance did not depend significantly on either body mass (both *p* = 0.13; Figure 4) or change in body mass (*p *> 0.3). All further results are provided for mass‐adjusted values.

**FIGURE 5:**
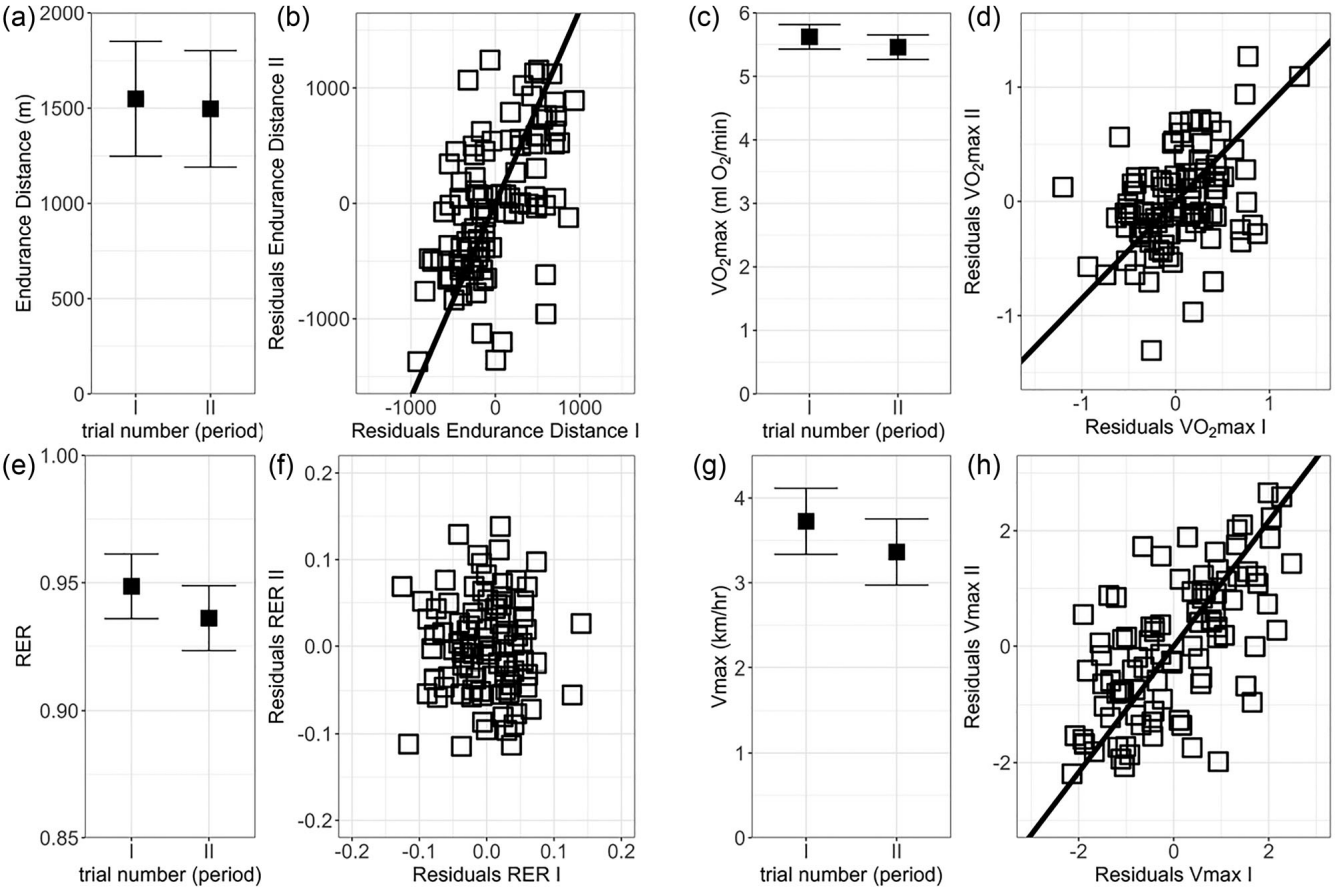
The repeatability of traits. (a) Mean endurance distance did not change between the two repeated trials (*p* = 0.4). (b) The endurance distances measured in the two repeated trials were correlated (*p *< 0.0001, *r *= 0.5). (c) Mean forced running aerobic capacity (${\dot{V}}_{O_{2}\max}$) was 25% lower in period II than in period I (*p* = 0.02). (d) The values of ${\dot{V}}_{O_{2}\max}$ measured in the two repeated trials were correlated (*p* = 0.0005, *r* = 0.4). (e) Mean RER did not change between the two repeated trials (*p* = 0.4). (f) Values of RER measured in the two repeated trials were not correlated (*p* = 0.7, *r* = −0.02). (g) Mean *V*_max_ did not significantly change between the two repeated trials (*p* = 0.07). (h) The values of *V*_max_ measured in the two repeated trials were correlated (*p *< 0.0001, *r* = 0.6). Note that, as explained in the legend to Figure 3, an overlap of the confidence limits does not indicate that a difference is not significant. See Table S4 for results of the proper significance tests. The graphs (a, c, e, g) show least‐squares means ± 95% confidence interval. The lines in graphs (b, d, h) show the reduced major axis regressions (not shown in graph f, because the values were not correlated). Abbreviations: RER, respiratory exchange ratio; *V*_max_, maximum speed achieved during the ${\dot{V}}_{O_{2}\max}$ trial.

The ${\dot{V}}_{O_{2}\max}$ was 2.9% lower in period II than period I (*p* = 0.008), but the other three traits did not differ significantly between the repeated trials (*p* ≥ 0.07). The residuals of ${\dot{V}}_{O_{2}\max}$, *V*_max_ and endurance distance measured in the two trials were correlated (0.4 ≤ *r* ≤ 0.6, *p *< 0.0005), but residuals of RER were not (*r* = −0.02, *p* = 0.7; Figure 5).

**FIGURE 6:**
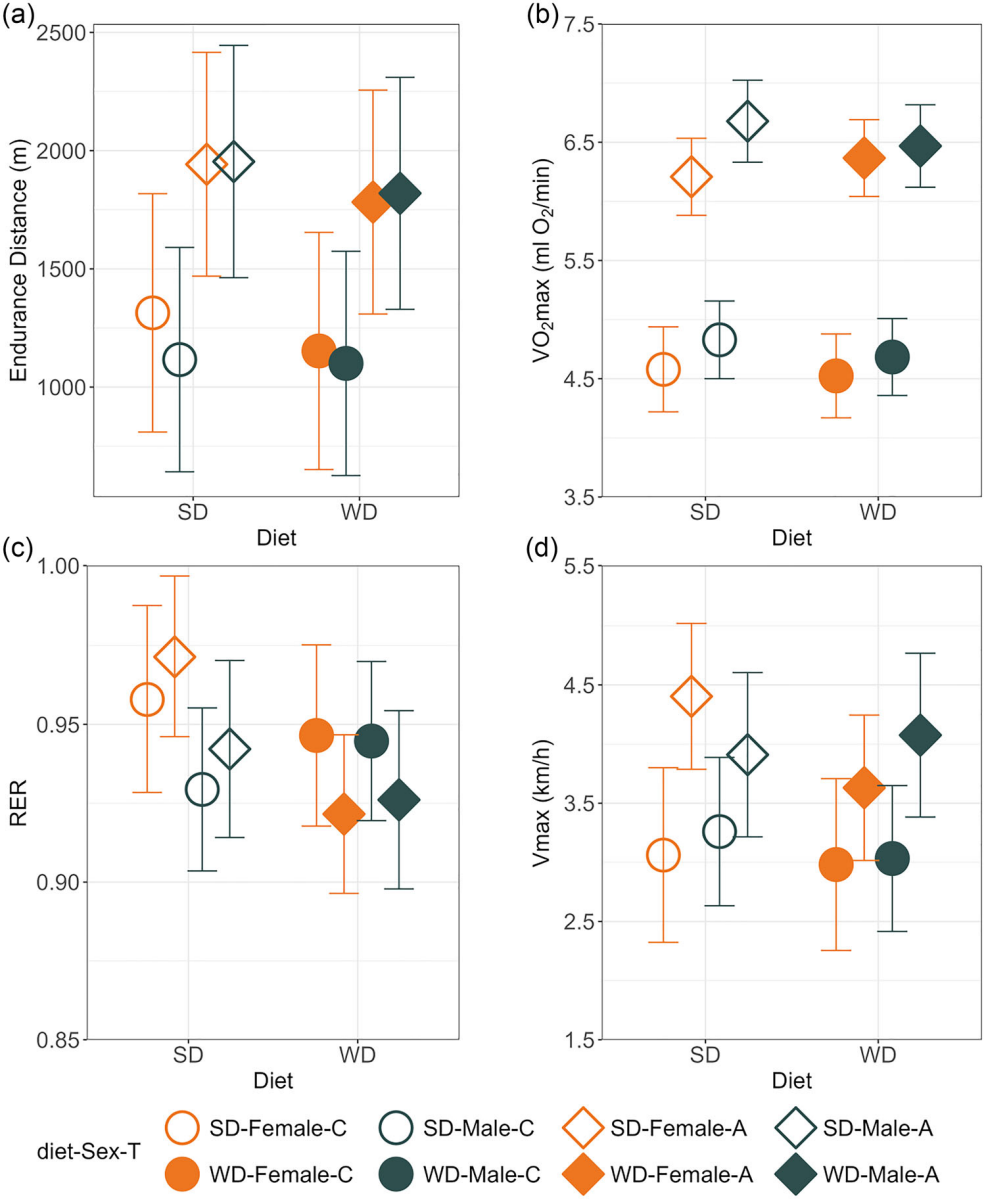
Effects of WD consumption on the locomotor and metabolic performance. (a) Mass‐adjusted endurance distance. (b) Mass‐adjusted forced running aerobic capacity (${\dot{V}}_{O_{2}\max}$). (c) Mass‐adjusted respiratory exchange ratio calculated at ${\dot{V}}_{O_{2}\max}$. (d) Mass‐adjusted maximum speed achieved during the ${\dot{V}}_{O_{2}\max}$ trial (*V*_max_). Note that, as explained in the legend to Figure 3, an overlap of the confidence limits does not indicate that a difference is not significant. See Table S4 for results of the proper significance tests. The graphs show least‐squares means ± 95% confidence interval. Abbreviations: A, aerobic lines; C, unselected control lines; SD, standard diet; WD, Western diet.

The endurance distance was 70% higher in A than in C lines (*p* = 0.02), and it decreased by ∼9% after the animals ate WD, but the diet effect was not significant (*p* = 0.099; Figure 6a). The effects of sex and diet × T interaction were not significant (*p* ≥ 0.7).

The ${\dot{V}}_{O_{2}\max}$ was 38% higher in A than in C lines (*p *< 0.0001; Figure 6b), but it was not affected by diet, sex or diet × T interaction (*p* ≥ 0.11). The RER did not differ consistently between the selection line types, diets or sexes (*p* ≥ 0.12), but the diet × T interaction was nearly significant (*p* = 0.07; Figure 6c). This was because in the SD conditions RER did not differ significantly between A and C lines (*p* = 0.36), and after feeding with WD it did not change in C lines (*p* = 0.85), whereas in A lines it decreased (*p* = 0.02).

The *V*_max_ was on average 30% higher in A than in C lines (*p* = 0.03), decreased on average 7% after eating WD (*p* = 0.04), and did not depend on sex (*p* = 0.86; Figure 6d). However, the pattern was complicated by interaction between the three factors (*p* = 0.01). The difference between the A and C lines was smaller after the animals ate WD (28%) than SD (32%), and the effect of diet differed between selection and sex groups. The *V*_max_ decreased after eating WD in A‐line females (18%, *p* = 0.0006), but no effect was observed in males or in either sex in C lines (*p* ≥ 0.3).

**FIGURE 7:**
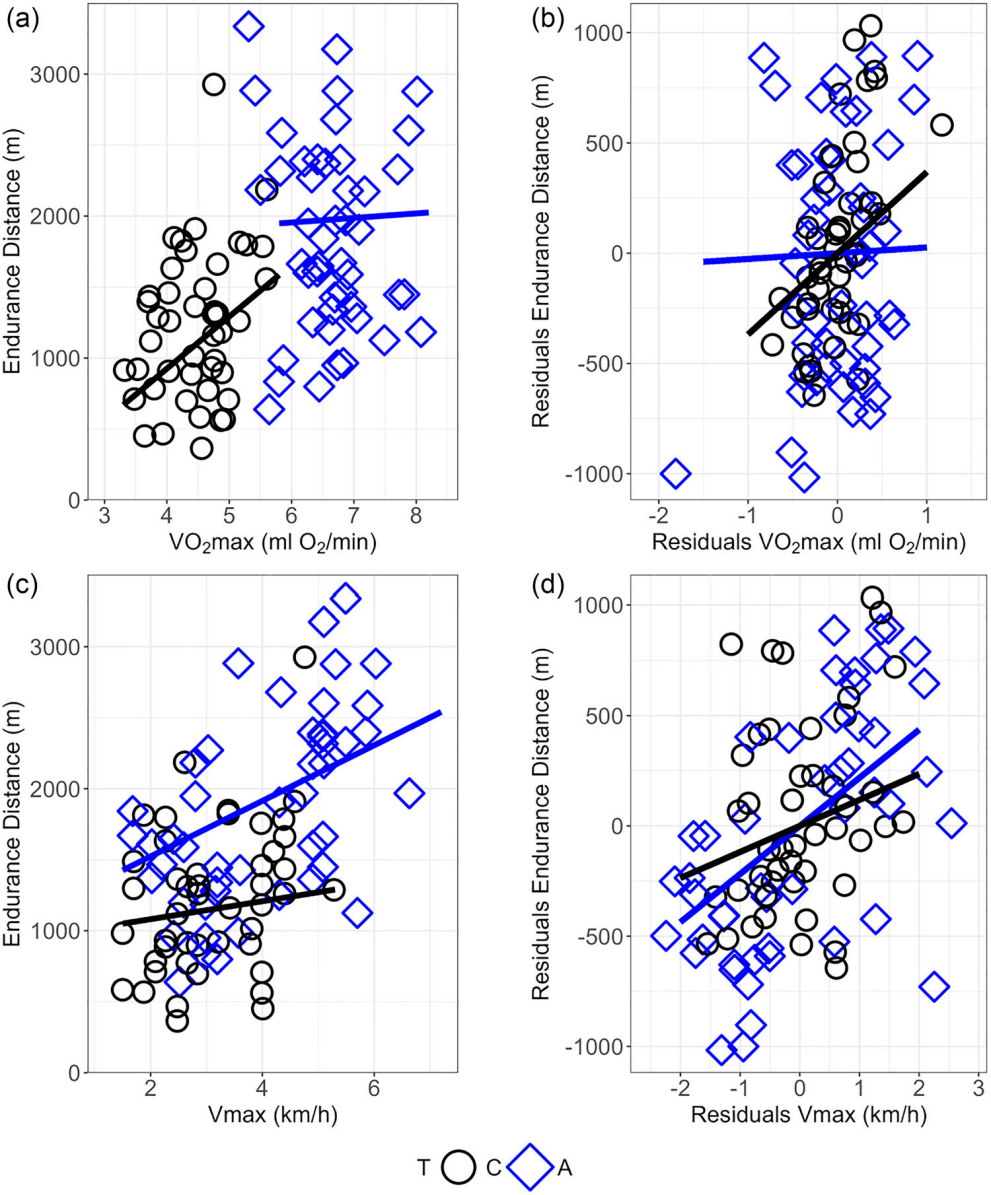
Correlation between the performance traits. The correlation between endurance distance and forced running aerobic capacity (${\dot{V}}_{O_{2}\max}$) (a, b) or maximum speed at ${\dot{V}}_{O_{2}\max}$ (*V*_max_) (c, d). (a, c) Graphs showing raw data. (b, d) Graphs showing residuals from final ANCOVA models. Slopes from the ANCOVA models are shown. Abbreviations: A, aerobic lines; C, unselected control lines.

The endurance distance on average increased with ${\dot{V}}_{O_{2}\max}$ (*p* = 0.014), but the relationship was complicated by a significant T × ${\dot{V}}_{O_{2}\max}$ interaction (*p* = 0.03; Figure 7a,b). This was because the endurance distance increased with ${\dot{V}}_{O_{2}\max}$ in C lines [partial regression slope ± 95% CI: 366 ± 251 m/(mL O_2_/min), *p* = 0.005, *r* = 0.6], but not in A lines [slope = 32 ± 176 m/(mL O_2_/min), *p* = 0.7, *r* = 0.19]. In contrast, the endurance distance increased with *V*_max_ consistently (*p *< 0.0001; Figure 7c,d), and the T ×* V*_max_ interaction was not significant [*p* = 0.08; in C lines, slope = 63 ± 198 m/(km/h), *p* = 0.3, *r* = 0.3; in A lines, slope = 217 ± 81 m/(km/h), *p *< 0.0001, *r* = 0.6].

### 3.3 Body mass loss during the locomotor tests

**FIGURE 8:**
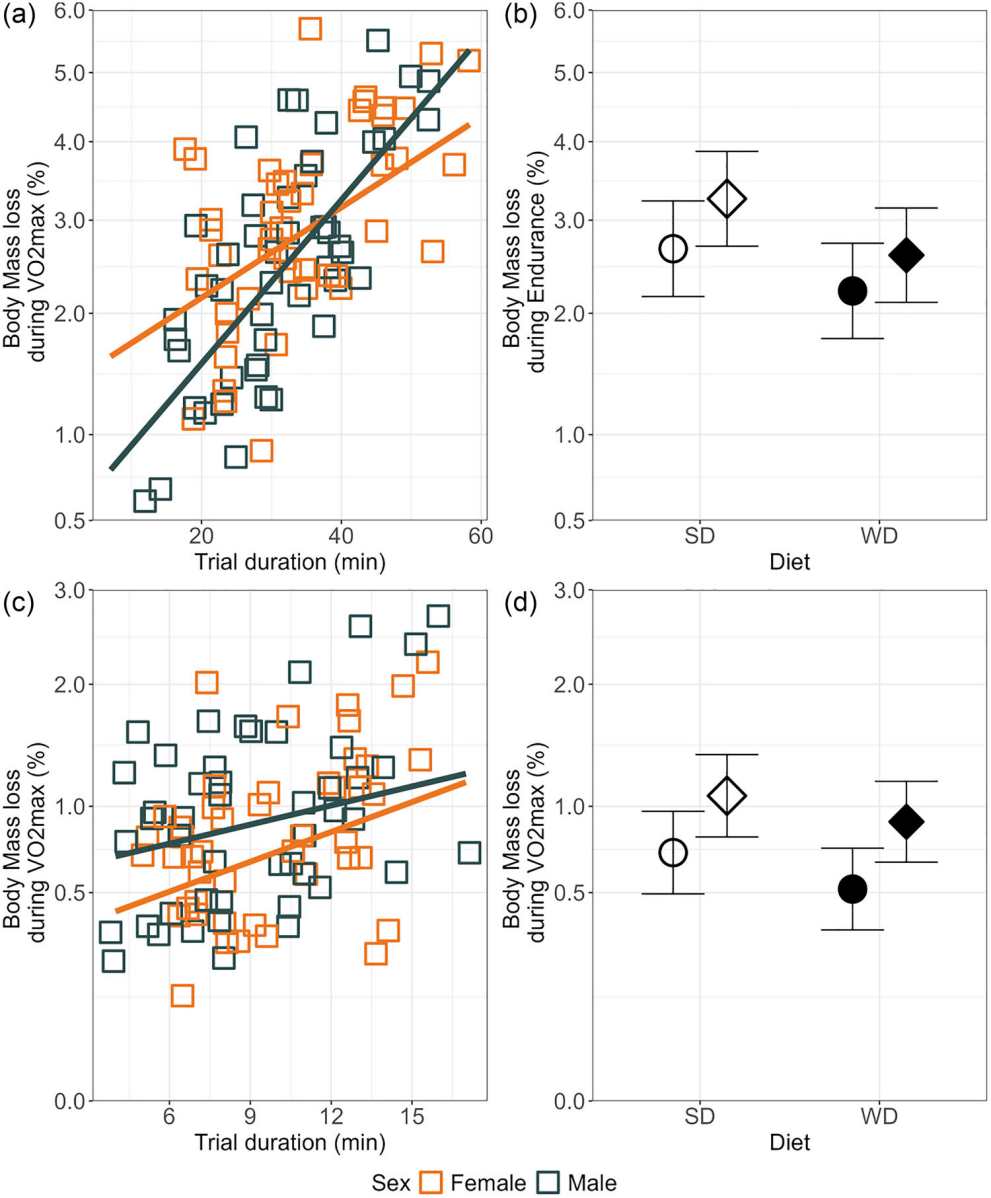
The percentage of body mass lost during trials. (a) Correlation between the percentage of body mass lost during endurance running and the trial duration. (b) The percentage of body mass lost during endurance running. Animals fed the WD lost less mass than those fed the SD [square root back‐transformed least‐square means with confidence limits; SD, 3% (2.6%–3.4%); WD, 2.4% (2.1%–2.8%); *p* = 0.008]. (c) Correlation between the percentage of body mass lost during forced running aerobic capacity (${\dot{V}}_{O_{2}\max}$) trial and the trial duration. (d) The percentage of body mass lost during ${\dot{V}}_{O_{2}\max}$. Animals fed the WD lost less mass than those fed the SD [back‐transformed least‐squares means with confidence limits; SD, 0.9% (0.7%–1%); WD, 0.7% (0.6%–0.9%); *p* = 0.11]. Note that, as explained in the legend to Figure 3, an overlap of the confidence limits does not indicate that a difference is not significant. See Table S4 for results of the proper significance tests. The *y*‐axis is square root back transformed. Slopes and least‐squares means are obtained from ANCOVA models. Abbreviations: A, aerobic lines; C, unselected control lines; SD, standard diet; WD, Western diet.

The voles lost on average 1% body mass (range 0%–4%) during the ${\dot{V}}_{O_{2}\max}$ trials and 2.8% mass (0%–8%) during the endurance running trials, and the mass loss increased with the duration of trials (*p* ≤ 0.0009; Figure 8a,c). After adjusting for variation in trial duration, initial body mass, and the change in body mass from initial body mass, the mass lost during the ${\dot{V}}_{O_{2}\max}$ trial was not significantly affected by sex (*p* = 0.16) or sex × trial duration interaction (*p* = 0.5), but during the endurance trial the sex × trial duration interaction was significant (*p* = 0.01); females lost more mass than males when the trial was short, but the trend was the opposite when it was long. The mass lost was larger in A than in C lines during ${\dot{V}}_{O_{2}\max}$ trials (*p* = 0.01), but not during the endurance running trials (*p* = 0.18). Animals fed WD lost ∼0.11% less mass during ${\dot{V}}_{O_{2}\max}$ trials (*p* = 0.11) and 0.2% less mass (*p* = 0.008) during the endurance trials than those fed SD (Figure 8b,d).

## 4 DISCUSSION

Voles from the A lines, which were selected for increased voluntary swim‐induced maximal aerobic metabolism (${\dot{V}}_{O_{2}\mathrm{swim}}$), not only achieved an 80% higher ${\dot{V}}_{O_{2}\mathrm{swim}}$ (Figure 1b), but also performed better in the forced exercise trials than voles from the unselected C lines (Figure 6). As shown also in our earlier work (Jaromin et al., 2019), animals from the A lines had 40% higher forced‐running ${\dot{V}}_{O_{2}\max}$ and 30% higher maximum speed achieved in this trial (*V*_max_). Here, we showed that A‐line animals also achieved a 69% higher endurance running distance. Thus, the selection experiment provides a suitable model to study how inherent differences in metabolic characteristics can alter the effects of diet on body mass and locomotor performance. We hypothesized that eating a diet high in both fat and sugar (WD) before exercise trials would increase endurance distance and ${\dot{V}}_{O_{2}\max}$ and that the effect would be larger in A than in C lines. However, WD consumption did not affect ${\dot{V}}_{O_{2}\max}$ and tended to decrease, rather than increase, endurance distance and *V*_max_, and resulted in loss of less mass during running, similarly in both the A and C lines.

The results of the repeated‐measures crossover design were consistent in both the treatment‐order groups and in both repeated periods. The average body mass was stable before the experiment, did not differ between the two treatment‐order groups and remained constant throughout the training period and the two main test periods. This indicates proper randomization of individuals and shows that the animals were at a stable metabolic status, and that 6 days of rest between the trials was sufficient to restore the condition of the animals. The ${\dot{V}}_{O_{2}\max}$, *V*_max_ and endurance distance had similar mean values in the repeated trials and were correlated between trials (*r* ≥ 0.4; Figure 5), that is, the measurements were repeatable (although RER values were poorly repeatable). Thus, the estimates of performance traits are reliable, and the lack of a positive effect of diet cannot be attributed to an inadequate methodology.

Although the aerobic capacity sets an upper limit on the level of persistent exercise, the overall performance in endurance trials can also be limited by other factors, both biomechanical and metabolic (Bassett, 2000). The *V*_max_ has appeared to be a better predictor of endurance running performance than ${\dot{V}}_{O_{2}\max}$, supposedly because *V*_max_ depends on both aerobic and anaerobic metabolism, in addition to muscle strength and power (Paavolainen et al., 2000; Stratton et al., 2009). Such a pattern was observed in A lines, where endurance distance was positively correlated with *V*_max_ but not with ${\dot{V}}_{O_{2}\max}$. However, in C lines the endurance distance was correlated more strongly with ${\dot{V}}_{O_{2}\max}$ than with *V*_max_. This indicates that the endurance distance can be limited by the aerobic capacity in the non‐athletic C‐line animals, but not in the aerobic‐athletic A‐line animals.

We hypothesized that substrate availability could limit the endurance performance, particularly in individuals with high aerobic capacity, and therefore that performance could be enhanced by eating a high‐energy Western diet (WD) for a short period before the trials. To our knowledge, no studies on humans can be compared directly with our experiment. However, a 5 km time trial was slower in participants who consumed WD than in those who consumed a Mediterranean diet for 4 days before the trial, which suggested a negative effect of WD (Baker et al., 2019). However, the effect of WD was not checked against the habitual diet of the participants, hence the results should be treated with caution. In our experiment, we checked the effect of eating WD for 2 days before the exercise trials with respect to standard diet conditions. Moreover, the experiment included groups of individuals with inherently distinct levels of the aerobic exercise capacity. We expected that WD would increase performance in the aerobic exercise trials and that the high‐aerobic A‐line voles would benefit more from the WD treatment. However, ${\dot{V}}_{O_{2}\max}$ was not affected by the dietary manipulation, and, contrary to the expectation, the endurance distance and *V*_max_ tended to decrease in the WD‐fed animals, similarly in A and C lines (Figure 6). Thus, the results did not support the hypothesis that energy substrates availability limit the maximum forced‐exercise aerobic metabolism or performance in endurance running trials. The body mass increased on average by 0.5 g (2%) after the voles ate WD. Thus, it could be argued that the negative effect of WD on *V*_max_ and endurance distance was merely attributable to carrying a larger weight, hence a larger effort during running at a given speed. However, the statistical model included the change in mass as a covariate, that is, it accounted for such a biomechanical mechanism. Thus, it seems that even such a short consumption of WD might have an adverse effect on the efficiency of metabolic processes. It is already well known that long‐term consumption of WD has detrimental health effects (Hruby & Hu, 2015). Our results also showed that, contrary to naive intuition, short‐term unrestricted consumption of such a diet provides no gain in aerobic exercise capacity and can even decrease the ability and therefore the motivation to exercise intensively.

Voles fed WD lost a lower proportion of mass during the running trials than those fed SD, and this difference was also present after adjusting for the trial duration and changes in body mass associated with the changes of diet. From the perspective of endurance sports, it seems that even if WD consumption decreases performance in a single run, the post‐run condition can be improved, which can be advantageous in extreme endurance tournaments lasting several days. On the contrary, from the perspective of public health, the result shows that a gain in mass after even an episodic consumption of WD is difficult to compensate by extended physical exercise. Individuals eating a balanced diet lost in the same exercise more mass than those that ate the WD diet. Thus, the results help us to understand why attempts to fight obesity by increasing physical activity, but without changing bad dietary habits, are less effective (Clark, 2015).

When the effects of diets enriched in either carbohydrates or fats are studied, the expected shifts in metabolized substrates are evident (Burke et al., 2002; Hawley & Leckey, 2015). In our experiment, both substrates were in excess, and although ${\dot{V}}_{O_{2}\max}$ did not change, it is not known whether the metabolized substrates changed. Overall, the RER averaged ∼0.93 (Figure 6). Thus, as in humans, carbohydrates are the main substrate metabolized at the peak aerobic effort in voles. The effects of selection groups and diet on RER are difficult to interpret, because they appear only as an interaction; after eating WD, the non‐athletic C‐line voles did not markedly change RER (hence, presumably, the proportion of metabolized substrates), whereas RER decreased in the high‐aerobic A‐line voles, indicating increased fat oxidation (Figure 6c). This is in line with results seen in rats selectively bred for high (HCR) and low (LCR) intrinsic aerobic endurance running capacity (Morris et al., 2014). The RER was not different between LCR and HCR in the standard diet group, but when rats were fed a high‐fat diet for 3 days, RER decreased in both LCR and HCR, but the decrease was larger in the HCR. Also, short‐term consumption (5 days) of a ketodiet by athletes before endurance running results in a fat adaptation, increased fat oxidation, hence carbohydrate protection (Burke et al., 2002; Hawley & Leckey, 2015). Although, as in the voles, the fat adaptation did not increase performance of the athletes running for ≤3 h, it could be beneficial in longer‐lasting, ultra‐endurance exercise (Burke et al., 2002). Notably, the adaptation appeared only in voles with genetically based increased aerobic capacity. Thus, the effects of supplementation of nutrients on performance traits depend on the genetic background, hence it is not surprising that outcomes of such manipulations in various groups of humans might be not consistent (Murphy et al., 2021; Wang et al., 2022).

The experiments on animal models have several advantages, but we should consider whether our model is relevant in the context of human nutritional and exercise physiology. Bank voles are omnivores, hence the nutritional composition of their diet fits within the wide scope of human diets. However, humans weigh ∼3000 times more than the voles. How does the effort of a 24 g vole compare to that of a 70 kg human? A reasonable approach to answer the question is to use heart rate as a physiological clock. Given that heart rate increases with body mass allometrically with a power of 0.25 (Günther & Morgado, 2005), the 38 min endurance running at a submaximal speed of an A‐line vole is equivalent to ∼4.5 h exercise for a human [(70 kg/0.024 kg)^0.25^ × 38 min], whereas 29 min running of a C‐line vole is equivalent to ∼3.5 h exercise for a human. The effort of the high‐aerobic athletic A‐line voles resembles that of ultra‐marathon runners, who covered the first 37 km of a run in ∼4.5 h and, like the voles, lose ∼3% of body mass (Gill et al., 2015). In contrast, the effort of the C‐line voles in the endurance trial is comparable to the effort in exercises undertaken to lose ∼2% of body mass, which is common in non‐athletes trying to maintain their physical fitness (Bellicha et al., 2021). Thus, the voles and the endurance running test protocol offer an adequate model to study factors affecting both exercise performance and body mass loss during exercise in individuals characterized by distinct metabolic characteristics.

## 5 CONCLUSION

In conclusion, bank voles from the selection experiment represent a suitable model for athletic and non‐athletic individuals with inherently distinct aerobic metabolic rates and endurance capacity, in which performance is likely to be limited by different physiological mechanisms. However, regardless of genetic background, consumption of the Western diet for 2 days before the trials did not increase aerobic capacity, tended to decrease endurance running distance and decreased mass loss during the exercise. Thus, short‐term consumption of the Western diet is not a promising enhancer of aerobic capacity and endurance performance, and it decreases the effectiveness of exercise as a method of combating obesity.

## AUTHOR CONTRIBUTIONS

Alaa Hseiky performed the research, acquired the data, analysed the data and wrote the original draft of the manuscript. All authors have conceived and designed the experiment, interpreted the data and contributed to the manuscript writing, reviewing and editing. All authors have read and approved the final version of the manuscript, agree to be accountable for all aspects of the work in ensuring that questions related to the accuracy or integrity of any part of the work are appropriately investigated and resolved. All persons designated as authors qualify for authorship, and all those who qualify for authorship are listed.

## CONFLICT OF INTEREST

None declared.

## Supporting information



Supporting Information

Supporting Information

## Data Availability

The datasets generated and analysed during the present study will be placed in the open repository RODBUK upon publication (https://doi.org/10.57903/UJ/EY8BWO).

## References

[eph70000-bib-0001] Acosta, W. , Meek, T. H. , Schutz, H. , Dlugosz, E. M. , & Garland, T. (2017). Preference for Western diet coadapts in High Runner mice and affects voluntary exercise and spontaneous physical activity in a genotype‐dependent manner. Behavioural Processes, 135, 56–65. 27908664 10.1016/j.beproc.2016.11.018

[eph70000-bib-0002] Areta, J. L. , & Hopkins, W. G. (2018). Skeletal muscle glycogen content at rest and during endurance exercise in humans: A meta‐analysis. Sports Medicine, 48(9), 2091–2102. 29923148 10.1007/s40279-018-0941-1

[eph70000-bib-0003] Baker, M. E. , DeCesare, K. N. , Johnson, A. , Kress, K. S. , Inman, C. L. , & Weiss, E. P. (2019). Short‐term Mediterranean diet improves endurance exercise performance: A randomized‐sequence crossover trial. Journal of the American College of Nutrition, 38(7), 597–605. 30758261 10.1080/07315724.2019.1568322

[eph70000-bib-0004] Bassett, D. R. (2000). Limiting factors for maximum oxygen uptake and determinants of endurance performance. Medicine & Science in Sports & Exercise, 32(1), 70–84. 10647532 10.1097/00005768-200001000-00012

[eph70000-bib-0005] Bellicha, A. , van Baak, M. A. , Battista, F. , Beaulieu, K. , Blundell, J. E. , Busetto, L. , Carraça, E. V. , Dicker, D. , Encantado, J. , Ermolao, A. , Farpour‐Lambert, N. , Pramono, A. , Woodward, E. , & Oppert, J. (2021). Effect of exercise training on weight loss, body composition changes, and weight maintenance in adults with overweight or obesity: An overview of 12 systematic reviews and 149 studies. Obesity Reviews, 22(S4), e13256. 33955140 10.1111/obr.13256 PMC8365736

[eph70000-bib-0006] Bloomer, R. J. , Schriefer, J. H. M. , Gunnels, T. A. , Lee, S.‐R. , Sable, H. J. , van der Merwe, M. , Buddington, R. K. , & Buddington, K. K. (2018). Nutrient intake and physical exercise significantly impact physical performance, body composition, blood lipids, oxidative stress, and inflammation in male rats. Nutrients, 10(8), 1109. 30126091 10.3390/nu10081109 PMC6115754

[eph70000-bib-0007] Boratyński, Z. , Szyrmer, M. , & Koteja, P. (2020). The metabolic performance predicts home range size of bank voles: A support for the behavioral‐bioenergetics theory. Oecologia, 193(3), 547–556. 32638120 10.1007/s00442-020-04704-x

[eph70000-bib-0008] Burke, L. M. , & Hawley, J. A. (2018). Swifter, higher, stronger: What's on the menu? Science (New York, N.Y.), 362(6416), 781–787. 30442803 10.1126/science.aau2093

[eph70000-bib-0009] Burke, L. M. , Hawley, J. A. , Angus, D. J. , Cox, G. R. , Clark, S. A. , Cummings, N. K. , Desbrow, B. , & Hargreaves, M. (2002). Adaptations to short‐term high‐fat diet persist during exercise despite high carbohydrate availability. Medicine and Science in Sports and Exercise, 34(1), 83–91. 11782652 10.1097/00005768-200201000-00014

[eph70000-bib-0010] Cao, J. , Lei, S. , Wang, X. , & Cheng, S. (2021). The effect of a ketogenic low‐carbohydrate, high‐fat diet on aerobic capacity and exercise performance in endurance athletes: A systematic review and meta‐analysis. Nutrients, 13(8), 2896. 34445057 10.3390/nu13082896 PMC8400555

[eph70000-bib-0011] Clark, J. E. (2015). Diet, exercise or diet with exercise: Comparing the effectiveness of treatment options for weight‐loss and changes in fitness for adults (18–65 years old) who are overfat, or obese; systematic review and meta‐analysis. Journal of Diabetes and Metabolic Disorders, 14(1), 31. 25973403 10.1186/s40200-015-0154-1 PMC4429709

[eph70000-bib-0012] Clemente, F. M. , Nikolaidis, P. T. , Martins, F. M. L. , & Mendes, R. S. (2016). Weekly physical activity patterns of university students: Are athletes more active than non‐athletes?. SpringerPlus, 5(1), 1808. 27812448 10.1186/s40064-016-3508-3 PMC5069237

[eph70000-bib-0013] Cordain, L. , Eaton, S. B. , Sebastian, A. , Mann, N. , Lindeberg, S. , Watkins, B. A. , O'Keefe, J. H. , & Brand‐Miller, J. (2005). Origins and evolution of the Western diet: Health implications for the 21st century. The American Journal of Clinical Nutrition, 81(2), 341–354. 15699220 10.1093/ajcn.81.2.341

[eph70000-bib-0014] Erdmann, J. , Wiciński, M. , Wódkiewicz, E. , Nowaczewska, M. , Słupski, M. , Otto, S. W. , Kubiak, K. , Huk‐Wieliczuk, E. , & Malinowski, B. (2021). Effects of energy drink consumption on physical performance and potential danger of inordinate usage. Nutrients, 13(8), 2506. 34444666 10.3390/nu13082506 PMC8401129

[eph70000-bib-0015] Erlenbusch, M. , Haub, M. , Munoz, K. , MacConnie, S. , & Stillwell, B. (2005). Effect of high‐fat or high‐carbohydrate diets on endurance exercise: A meta‐analysis. International Journal of Sport Nutrition and Exercise Metabolism, 15(1), 1–14. 15902985 10.1123/ijsnem.15.1.1

[eph70000-bib-0016] Eslami, O. , Zarei, M. , & Shidfar, F. (2020). The association of dietary patterns and cardiorespiratory fitness: A systematic review. Nutrition, Metabolism, and Cardiovascular Diseases: NMCD, 30(9), 1442–1451. 32513576 10.1016/j.numecd.2020.04.017

[eph70000-bib-0017] Garton, F. C. , North, K. N. , Koch, L. G. , Britton, S. L. , Nogales‐Gadea, G. , & Lucia, A. (2016). Rodent models for resolving extremes of exercise and health. Physiological Genomics, 48(2), 82–92. 26395598 10.1152/physiolgenomics.00077.2015 PMC4729696

[eph70000-bib-0018] Gill, S. K. , Teixeira, A. , Rama, L. , Prestes, J. , Rosado, F. , Hankey, J. , Scheer, V. , Hemmings, K. , Ansley‐Robson, P. , & Costa, R. J. S (2015). Circulatory endotoxin concentration and cytokine profile in response to exertional‐heat stress during a multi‐stage ultra‐marathon competition. Exercise Immunology Review, 21, 114–128. 25830597

[eph70000-bib-0019] Günther, B. , & Morgado, E. (2005). Allometric scaling of biological rhythms in mammals. Biological Research, 38(2–3), 207–212. 16238099 10.4067/s0716-97602005000200010

[eph70000-bib-0020] Hawley, J. A. , & Leckey, J. J. (2015). Carbohydrate dependence during prolonged, intense endurance exercise. Sports Medicine (Auckland, N.Z.), 45(S1), 5–12. 10.1007/s40279-015-0400-1 PMC4672006 26553495

[eph70000-bib-0021] Helge, J. W. , Ayre, K. , Chaunchaiyakul, S. , Hulbert, A. J. , Kiens, B. , & Storlien, L. H. (1998). Endurance in high‐fat‐fed rats: Effects of carbohydrate content and fatty acid profile. Journal of Applied Physiology, 85(4), 1342–1348. 9760326 10.1152/jappl.1998.85.4.1342

[eph70000-bib-0022] Henderson, N. D. (1997). Spurious associations in unreplicated selected lines. Behavior Genetics, 27(2), 145–154. 9145553 10.1023/a:1025689425738

[eph70000-bib-0023] Hoppeler, H. , & Weibel, E. R. (1998). Limits for oxygen and substrate transport in mammals. The Journal of Experimental Biology, 201(8), 1051–1064. 9510519 10.1242/jeb.201.8.1051

[eph70000-bib-0024] Hruby, A. , & Hu, F. B. (2015). The epidemiology of obesity: A big picture. PharmacoEconomics, 33(7), 673–689. 25471927 10.1007/s40273-014-0243-x PMC4859313

[eph70000-bib-0025] Hseiky, A. , Lipowska, M. M. , Sadowska, E. T. , Józkowicz, A. , Nowak, W. N. , & Koteja, P. (2024). Effect of Western diet on body composition, locomotor performance and blood biochemical profile in the bank vole. *bioRxiv*. 10.1101/2024.11.20.624415 40931831

[eph70000-bib-0026] Hu, J. , Wang, Z. , Lei, B. , Li, J. , & Wang, R. (2021). Effects of a low‐carbohydrate high‐fat diet combined with high‐intensity interval training on body composition and maximal oxygen uptake: A systematic review and meta‐analysis. International Journal of Environmental Research and Public Health, 18(20), 10740. 34682481 10.3390/ijerph182010740 PMC8535842

[eph70000-bib-0027] Jansson, A. , & Harris, P. A. (2013). A bibliometric review on nutrition of the exercising horse from 1970 to 2010. Comparative Exercise Physiology, 9(3‐4), 169–180.

[eph70000-bib-0028] Jaromin, E. , Sadowska, E. T. , & Koteja, P. (2019). Is experimental evolution of an increased aerobic exercise performance in bank voles mediated by endocannabinoid signaling pathway? Frontiers in Physiology, 10, 640. 31191344 10.3389/fphys.2019.00640 PMC6546880

[eph70000-bib-0029] Jaromin, E. , Wyszkowska, J. , Labecka, A. M. , Sadowska, E. T. , & Koteja, P. (2016). Hindlimb muscle fibre size and glycogen stores in bank voles with increased aerobic exercise metabolism. The Journal of Experimental Biology, 219(Pt 4), 470–473. 26685167 10.1242/jeb.130476

[eph70000-bib-0030] Koch, L. G. , & Britton, S. L. (2001). Artificial selection for intrinsic aerobic endurance running capacity in rats. Physiological Genomics, 5(1), 45–52. 11161005 10.1152/physiolgenomics.2001.5.1.45

[eph70000-bib-0031] Koteja, P. (1996). Measuring energy metabolism with open‐flow respirometric systems: Which design to choose? Functional Ecology, 10(5), 675.

[eph70000-bib-0032] Koteja, P. , Baliga‐Klimczyk, K. , Chlad, A. , Chrzascik, K. M. , Damulewicz, M. , Dragosz‐Kluska, D. , Morawska‐Ploskonka, J. , & Sadowska, E. T. (2009). Correlated responses to a multidirectional artificial selection in the bank vole: Activity, metabolism, and food consumption. XXXVIth International Congress of Physiological Sciences (IUPS‐2009), 27/07‐1/08/2009, Kioto, Japan.

[eph70000-bib-0033] Koteja, P. , Baliga‐Klimczyk, K. , ChrzĄścik, K. M. , Dheyongera, G. , Rudolf, A. , Sumicka, S. , & Sadowska, E. T. (2011). Experimental evolution of metabolic rates: Correlated responses to a multidirectional artificial selection in the bank vole, *Myodes glareolus* . *Journal of Physiology and Pharmacology*, 62s1:212 (25th Congress of the Polish Physiological Society, September 15‐17, 2011, Olsztyn).

[eph70000-bib-0034] Lighton, J. R. B. (2008). Measuring metabolic rates: A manual for scientists. Oxford University Press. 10.1093/acprof:oso/9780195310610.001.0001

[eph70000-bib-0035] Maroofi, A. , Bagheri Rouch, A. , Naderi, N. , & Damirchi, A. (2022). Effects of two different exercise paradigms on cardiac function, BDNF‐TrkB expression, and myocardial protection in the presence and absence of Western diet. International Journal of Cardiology‐Heart & Vasculature, 40, 101022. 35399608 10.1016/j.ijcha.2022.101022 PMC8991101

[eph70000-bib-0036] Meek, T. H. , Dlugosz, E. M. , Vu, K. T. , & Garland, T. (2012). Effects of leptin treatment and Western diet on wheel running in selectively bred high runner mice. Physiology & Behavior, 106(2), 252–258. 22361262 10.1016/j.physbeh.2012.02.012

[eph70000-bib-0037] Meek, T. H. , Eisenmann, J. C. , & Garland, T. (2010). Western diet increases wheel running in mice selectively bred for high voluntary wheel running. International Journal of Obesity, 34(6), 960–969. 20157317 10.1038/ijo.2010.25

[eph70000-bib-0038] Morris, E. M. , Jackman, M. R. , Johnson, G. C. , Liu, T.‐W. , Lopez, J. L. , Kearney, M. L. , Fletcher, J. A. , Meers, G. M. E. , Koch, L. G. , Britton, S. L. , Rector, R. S. , Ibdah, J. A. , MacLean, P. S. , & Thyfault, J. P. (2014). Intrinsic aerobic capacity impacts susceptibility to acute high‐fat diet‐induced hepatic steatosis. American Journal of Physiology—Endocrinology and Metabolism, 307(4), E355–E364. 24961240 10.1152/ajpendo.00093.2014 PMC4137118

[eph70000-bib-0039] Murphy, N. E. , Carrigan, C. T. , & Margolis, L. M. (2021). High‐fat ketogenic diets and physical performance: A systematic review. Advances in Nutrition (Bethesda, Md.), 12(1), 223–233. 32865567 10.1093/advances/nmaa101 PMC7850028

[eph70000-bib-0040] Murray, A. J. , Knight, N. S. , Cochlin, L. E. , McAleese, S. , Deacon, R. M. J. , Rawlins, J. N. P. , & Clarke, K. (2009). Deterioration of physical performance and cognitive function in rats with short‐term high‐fat feeding. Federation of American Societies for Experimental Biology Journal, 23(12), 4353–4360. 19667117 10.1096/fj.09-139691

[eph70000-bib-0041] Ormsbee, M. J. , Bach, C. W. , & Baur, D. A. (2014). Pre‐exercise nutrition: The role of macronutrients, modified starches and supplements on metabolism and endurance performance. Nutrients, 6(5), 1782–1808. 24787031 10.3390/nu6051782 PMC4042570

[eph70000-bib-0042] Paavolainen, L. , Nummela, A. , & Rusko, H. (2000). Muscle power factors and VO2max as determinants of horizontal and uphill running performance. Scandinavian Journal of Medicine & Science in Sports, 10(5), 286–291. 11001396 10.1034/j.1600-0838.2000.010005286.x

[eph70000-bib-0043] Phelps, N. H. , Singleton, R. K. , Zhou, B. , Heap, R. A. , Paciorek, C. J. , Lhoste, V. P. , Carrillo‐Larco, R. M. , Stevens, G. A. , Rodriguez‐Martinez, A. , Bixby, H. , Bentham, J. , Di Cesare, M. , Danaei, G. , Rayner, A. W. , Barradas‐Pires, A. , Cowan, M. J. , Savin, S. , Riley, L. M. , Aguilar‐Salinas, C. A. , … Ezzati, M. (2024). Worldwide trends in underweight and obesity from 1990 to 2022: A pooled analysis of 3663 population‐representative studies with 222 million children, adolescents, and adults. The Lancet, 403(10431), 1027–1050. 10.1016/S0140-6736(23)02750-2 PMC7615769 38432237

[eph70000-bib-0044] Ronto, p. (2020). The State of Ultra Running 2020. RunRepeat—Athletic Shoe Reviews. https://runrepeat.com/state‐of‐ultra‐running

[eph70000-bib-0045] Ross, R. , Goodpaster, B. H. , Koch, L. G. , Sarzynski, M. A. , Kohrt, W. M. , Johannsen, N. M. , Skinner, J. S. , Castro, A. , Irving, B. A. , Noland, R. C. , Sparks, L. M. , Spielmann, G. , Day, A. G. , Pitsch, W. , Hopkins, W. G. , & Bouchard, C. (2019). Precision exercise medicine: Understanding exercise response variability. British Journal of Sports Medicine, 53(18), 1141–1153. 30862704 10.1136/bjsports-2018-100328 PMC6818669

[eph70000-bib-0046] Sadowska, E. T. , Baliga‐Klimczyk, K. , Chrzaścik, K. M. , & Koteja, P. (2008). Laboratory model of adaptive radiation: A selection experiment in the bank vole. Physiological and Biochemical Zoology : PBZ, 81(5), 627–640. 18781839 10.1086/590164

[eph70000-bib-0047] Sadowska, E. T. , Stawski, C. , Rudolf, A. , Dheyongera, G. , Chrząścik, K. M. , Baliga‐Klimczyk, K. , & Koteja, P. (2015). Evolution of basal metabolic rate in bank voles from a multidirectional selection experiment. Proceedings‐Biological Sciences, 282(1806), 20150025. 25876844 10.1098/rspb.2015.0025 PMC4426621

[eph70000-bib-0048] Sadowska, J. , Gębczyński, A. K. , & Konarzewski, M. (2017). Selection for high aerobic capacity has no protective effect against obesity in laboratory mice. Physiology & Behavior, 175, 130–136. 28363839 10.1016/j.physbeh.2017.03.034

[eph70000-bib-0049] Scheer, V. , Ramme, K. , Reinsberger, C. , & Heitkamp, H.‐C. (2018). VO2max testing in trail runners: Is there a specific exercise test protocol?. International Journal of Sports Medicine, 39(06), 456–461. 29665614 10.1055/a-0577-4851

[eph70000-bib-0050] Schipke, J. , Vital, M. , Schnapper‐Isl, A. , Pieper, D. H. , & Mühlfeld, C. (2019). Spermidine and voluntary activity exert differential effects on sucrose‐ compared with fat‐induced systemic changes in male mice. The Journal of Nutrition, 149(3), 451–462. 30715385 10.1093/jn/nxy272

[eph70000-bib-0051] Smith, N. J. , Caldwell, J. L. , van der Merwe, M. , Sharma, S. , Butawan, M. , Puppa, M. , & Bloomer, R. J. (2019). A comparison of dietary and caloric restriction models on body composition, physical performance, and metabolic health in young mice. Nutrients, 11(2), 350. 30736418 10.3390/nu11020350 PMC6412800

[eph70000-bib-0052] Srivastava, S. , Tamrakar, S. , Nallathambi, N. , Vrindavanam, S. A. , Prasad, R. , & Kothari, R. (2024). Assessment of maximal oxygen uptake (VO2max) in athletes and nonathletes assessed in sports physiology laboratory. Cureus, 16(5), e61124. 38919211 10.7759/cureus.61124 PMC11197041

[eph70000-bib-0053] Stratton, E. , O'Brien, B. J. , Harvey, J. , Blitvich, J. , McNicol, A. J. , Janissen, D. , Paton, C. , & Knez, W. (2009). Treadmill velocity best predicts 5000‐m run performance. International Journal of Sports Medicine, 30(01), 40–45. 19202577 10.1055/s-2008-1038761

[eph70000-bib-0054] Swallow, J. , Hayes, J. , Koteja, P. , & Garland, T. (2009). Selection experiments and experimental evolution of performance and physiology. In T. Garland Jr. , & M. R. Rose (Eds.), Experimental Evolution: Concepts, Methods, and Applications of Selection Experiments (pp. 301–351). University of California, Berkeley, CA. 10.1525/california/9780520247666.003.0012

[eph70000-bib-0055] Vitale, K. , & Getzin, A. (2019). Nutrition and supplement update for the endurance athlete: Review and recommendations. Nutrients, 11(6), 1289. 31181616 10.3390/nu11061289 PMC6628334

[eph70000-bib-0056] Wang, Y. , Zhou, K. , Wang, V. , Bao, D. , & Zhou, J. (2022). The effects of concurrent training combined with low‐carbohydrate high‐fat ketogenic diet on body composition and aerobic performance: A systematic review and meta‐analysis. International Journal of Environmental Research and Public Health, 19(18), 11542. 36141816 10.3390/ijerph191811542 PMC9517144

[eph70000-bib-0057] Xiao, Y. , Wang, W. , Chen, L. , Chen, J. , Jiang, P. , Fu, X. , Nie, X. , Kwan, H. , Liu, Y. , & Zhao, X. (2017). The effects of short‐term high‐fat feeding on exercise capacity: Multi‐tissue transcriptome changes by RNA sequencing analysis. Lipids in Health and Disease, 16(1), 28. 28153015 10.1186/s12944-017-0424-7 PMC5290644

[eph70000-bib-0058] Yen, C. H. , Tsao, T. H. , Huang, C. U. , Yang, C. B. , & Kuo, C. S. (2013). Effects of sweet cassava polysaccharide extracts on endurance exercise in rats. Journal of the International Society of Sports Nutrition, 10(1), 18. 23537169 10.1186/1550-2783-10-18 PMC3620959

